# Dr. A. W. Allen

**Published:** 1869-02

**Authors:** 


Dr. A. W. Allen.—From Prof. W. H. Atkinson we regret to learn of the death of this gentleman, a brother of Dr. W. H. Allen of 18 W. 11th St., N.Y. Dr. Allen died suddenly on the evening of Jan. 8th; at 3 P.M. of this day when engaged in operating, he was suddenly seized with a severe pain in the head and at 6.40 was a corpse. His disease was denominated apoplexy.

